# State-Dependent CNN–GRU Reinforcement Framework for Robust EEG-Based Sleep Stage Classification

**DOI:** 10.3390/biomimetics11010054

**Published:** 2026-01-08

**Authors:** Sahar Zakeri, Somayeh Makouei, Sebelan Danishvar

**Affiliations:** 1 Faculty of Electrical and Computer Engineering, University of Tabriz, Tabriz 51666-15813, Iran; zakeri@tabrizu.ac.ir; 2 College of Engineering, Design and Physical Sciences, Brunel University London, Uxbridge UB83PH, UK

**Keywords:** auditory stimuli, electroencephalography, Lempel–Ziv complexity, microstates, reinforcement learning, sleep

## Abstract

Recent advances in automated learning techniques have enhanced the analysis of biomedical signals for detecting sleep stages and related health abnormalities. However, many existing models face challenges with imbalanced datasets and the dynamic nature of evolving sleep states. In this study, we present a robust algorithm for classifying sleep states using electroencephalogram (EEG) data collected from 33 healthy participants. We extracted dynamic, brain-inspired features, such as microstates and Lempel–Ziv complexity, which replicate intrinsic neural processing patterns and reflect temporal changes in brain activity during sleep. An optimal feature set was identified based on significant spectral ranges and classification performance. The classifier was developed using a convolutional neural network (CNN) combined with gated recurrent units (GRUs) within a reinforcement learning framework, which models adaptive decision-making processes similar to those in biological neural systems. Our proposed biomimetic framework illustrates that a multivariate feature set provides strong discriminative power for sleep state classification. Benchmark comparisons with established approaches revealed a classification accuracy of 98% using the optimized feature set, with the framework utilizing fewer EEG channels and reducing processing time, underscoring its potential for real-time deployment. These findings indicate that applying biomimetic principles in feature extraction and model design can improve automated sleep monitoring and facilitate the development of novel therapeutic and diagnostic tools for sleep-related disorders.

## 1. Introduction

Sleep is a fundamental biological state that underpins numerous physiological and cognitive functions, including energy restoration, emotional stability, and memory processing [1,2,3]. In recent decades, neuroscience has emphasized that sleep is not a passive loss of awareness but an active condition characterized by dynamic neural activity [4]. Different stages of sleep—non-rapid eye movement (NREM) and rapid eye movement (REM)—contribute uniquely to memory systems [5]. NREM sleep is largely associated with the consolidation of declarative memories, whereas REM sleep plays a critical role in emotional and procedural memory. Despite these findings, the underlying neural mechanisms remain only partially understood, particularly when external sensory inputs interact with these processes.

Auditory stimulation, and music in particular, has emerged as a promising non-invasive method for modulating sleep-related brain activity. Previous studies report that music can benefit individuals with sleep disturbances. Presenting sound during sleep has drawn attention because it can influence neural activity without necessarily altering sleep architecture. When delivered in optimal phases, auditory cues have been shown to enhance memory consolidation through target memory reactivation [6,7]. However, most prior research relied on simple tones or word cues [8,9,10]. The potential of complex, emotionally engaging auditory inputs—such as music—remains relatively underexplored as a therapeutic tool. Music contains rich rhythmic, melodic, and affective elements that may activate brain networks differently than simple sounds [6]. Exploring its influence on brain oscillations, state transitions, and temporal diversity during sleep could clarify how auditory information is processed in the unconscious state and whether it can actively shape neural acuity for therapeutic applications.

Electroencephalography (EEG) provides insight into the oscillatory and network-level dynamics underlying sleep [11]. During quiet wakefulness with eyes closed, alpha activity (8–12 Hz) dominates [12]. As sleep begins, EEG activity slows, with theta rhythms (4–7 Hz) prevalent in NREM stage 1, alongside k-complexes and spindles (12–15 Hz) emerging in NREM stage 2 [13,14]. NREM stage 3 is characterized by slow, high-amplitude delta waves (<4 Hz), while REM sleep displays low-amplitude, mixed-frequency activity, resembling both light sleep and wake states.

In parallel, machine learning and deep learning methods have become powerful frameworks for the automated analysis of biomedical signals. These approaches offer robust tools for large-scale data analysis and have been widely applied to neurological research [15,16]. A variety of algorithms have been used for automated sleep stage classification, including Random Forest [17], Support Vector Machine [18,19,20], artificial neural networks [21], convolutional neural networks (CNNs) [22,23,24,25], recurrent neural networks (RNNs) [26], gated recurrent units (GRUs) [27], and graph-informed convolutional autoencoders (GICAs) [28]. While handcrafted features can provide domain-specific insights and interpretability [29,30], they often lack generalizability. Deep learning models, in contrast, can learn directly from raw data, improving adaptability across datasets [31,32].

Reinforcement learning (RL), as a subset of machine learning methods, enables an agent to learn sequential decision-making in dynamic environments to maximize cumulative rewards. Traditionally, RL has been primarily applied in robotics and autonomous systems, which require complex sequential decision-making. More recently, deep RL principles have been successfully leveraged in various domains such as investment strategy optimization [33], clinical decision-making frameworks for treatment planning [34], and energy optimization systems [35]. Building on these advances, recent studies have begun to extend RL methodologies to neuroscience, particularly in EEG and brain–computer interface (BCI) applications, where dynamic adaptation and sequential feedback are also essential. Girdler et al. [36] provided a technical overview of RL decoders in brain–machine interface systems. Aung et al. [37] introduced an EEG RL-Net for motor imagery classification, while Zhang et al. [38] developed an attention-based RL framework for EEG signal processing. Similarly, Fidêncio et al. [39] employed error-related potentials to design adaptive reward mechanisms in RL-driven BCIs. These studies collectively highlight the potential of RL to enhance adaptability, reward-guided learning, and feature optimization in EEG-based classification, thereby motivating the development of our RL-based framework for sleep state analysis.

The current study investigates how music played during REM sleep affects EEG spectral activity and temporal diversity. This work is based on the idea that external stimuli can trigger brain-like responses. These responses show how dynamic and self-organizing neural systems are. By examining brain activity before and after musical stimulation, we aim to determine if sound during REM can change natural neural rhythms and temporal complexity, similarly to how the brain reorganizes itself for memory and emotional processing. We hypothesize that hearing auditory exposure during REM sleep changes oscillatory patterns and increases variability across different brain regions. This reflects how the brain processes information in a way that follows the biomimetics idea. Additionally, we use multivariate classification models to identify differences between pre- and post-stimulation states. These models take advantage of systems that mimic the hierarchical and temporal processing seen in real biological networks. This study offers initial evidence that music-based stimulation during sleep could act as a non-invasive method to improve cognitive and emotional functions through the brain’s sleep-related mechanisms.

## 2. Materials and Methods

In this study, two datasets were utilized to evaluate the performance and generalizability of the proposed model. The first dataset was experimentally recorded by our team and includes EEG data under four representative conditions: wakefulness (eyes open), NREM sleep (eyes closed), REM sleep with auditory stimuli, and REM sleep without stimuli. This dataset was designed to investigate the neural effects of sound presentation during REM sleep. The following subsections describe the details of our experimentally recorded dataset.

The second dataset was obtained from an available source [40] and contains EEG recordings from healthy participants without any reported sleep or physiological disorders. This dataset follows the standard AASM scoring criteria and includes five sleep stages: wake, N1, N2, N3, and REM. To maintain methodological consistency, identical preprocessing and feature extraction procedures were applied to both datasets, and the same model architecture was used for classification [40].

### 2.1. Participants, Experimental Setup, and Protocol

To explore how sound stimuli impact human sleep, a group of healthy volunteers with a regular tendency to stay awake late was chosen. In total, 36 participants (33 right-handed and 3 left-handed) were involved, all without a history of psychological disorders or medication usage. Three participants were eliminated from further analysis due to waking up during the experiment. The data from the remaining participants (14 males; average age = 31.06 ± 13.75 years) were used for further analysis. This study was conducted at the Biomedical Engineering Laboratory of the University of Tabriz in Iran. All participants gave informed consent and filled out a health questionnaire.

### 2.2. Data Recording Protocol

All participants reported maintaining a habitual sleep duration of at least 7 h per night and were instructed to adhere to their regular sleep schedule prior to the study day. According to the scoring criteria set by the American Academy of Sleep Medicine (AASM), sleep is categorized into five stages: wakefulness, non-rapid eye movement (NREM), and rapid eye movement (REM). This research concentrated on four specific conditions: (a) wakefulness or eyes-open (EO), which was recorded for 5 min while participants were lying down with their eyes open; (b) NREM or eyes-closed (EC), recorded for 5 min as participants had their eyes closed and were transitioning into NREM sleep; (c) REM sleep with stimuli, recorded for 20 min during REM sleep accompanied by auditory stimuli; (d) REM sleep without stimuli, for a 5 min segment of REM sleep without any external input.

In the wakefulness state, EEG typically showcases predominant alpha activity (8–13 Hz), especially in the occipital regions, along with sporadic low-amplitude, mixed-frequency patterns. During NREM sleep, distinctive characteristics include diminished alpha power, the emergence of theta rhythms, sleep spindles (12–16 Hz), and high-amplitude delta activity during the transition from light to deep sleep [41]. Conversely, REM sleep is characterized by low-voltage, mixed-frequency signals, rapid eye movements, reduced muscle tone, and notable theta oscillations, resulting in desynchronized EEG patterns. The identification of sleep stages was conducted by integrating three EEG frequency-band characteristics in accordance with AASM guidelines. All sleep scoring was completed offline by a qualified sleep specialist. All participants were comfortably reclined on a bed within a sound-attenuated, dimly lit environment (illuminance < 50 Lux), maintained at roughly 25 °C. Mindfulness meditation techniques were utilized to aid the onset of sleep, which included lying in a comfortable position, minimizing distractions, and concentrating on their breathing (inhale for a count of 10, hold, exhale for a count of 10, and repeat this for ten cycles). No pharmacological substances were administered to ensure the neutral course of the sleep cycles.

**Figure 1:**
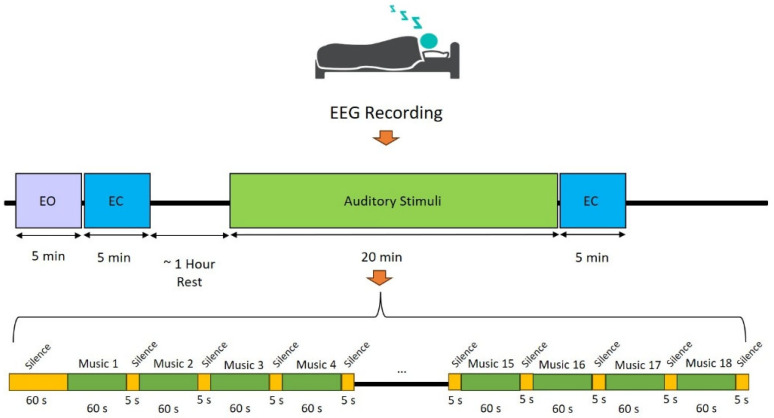
Illustration of the experimental procedure. Each recording session began with a 5 min eyes-open (EO) condition to establish a baseline of wakeful EEG activity. This was followed by an eyes-closed (EC) period corresponding to the onset of NREM sleep. After approximately one hour, upon identifying the REM sleep stage, auditory stimuli were presented to the participants. Subsequently, after a sufficient interval, an additional REM sleep segment was recorded without auditory stimulation. This sequence was constantly applied to all participants to ensure uniformity in sleep stage transitions and experimental conditions.

A varied array of auditory stimuli was employed, featuring both instrumental sounds (such as piano, hang drum, guitar, saxophone, santoor, kamancheh, tar, and violin) and natural sounds (including thunderstorm, forest rain, bird songs, ocean waves, crackling fire, and whale calls) [28]. To minimize habituation effects, each sound was presented exclusively during the REM stage. The stimuli were delivered via in-ear headphones (Apple, 3.5 mm wired earbuds), ensuring consistent sound presentation during sleep. The presentation procedure was uniform across all participants: a 60 s silent baseline preceding the first stimulus, followed by individual sound trials separated by 5 s silent intervals using Wave Pad Sound Editor [42]. Each stimulus lasted 60 s, resulting in a total exposure time of 1080 s (Figure 1). Auditory stimuli were played binaurally at an intensity of 45 dB SPL, a level selected to reduce sleep disruption while still ensuring perceptual significance. Sleep disruption was defined as significant changes in sleep continuity or architecture, such as abrupt transitions between sleep stages, brief EEG arousal lasting more than 3 s, or extended periods of wakefulness. Previous studies have shown that sound levels below 50 dB SPL typically do not cause major disturbances in sleep [43,44]. Therefore, 45 dB SPL is appropriate to detect changes in sleep dynamics without increasing arousal frequency or wakefulness. In this study, auditory stimuli were delivered solely during REM sleep, a phase known for intense dreaming and increased neural activity in areas associated with sensory processing [45,46,47]. Introducing sounds during REM enables the exploration of how the brain interprets external auditory information without rousing the individual, as well as examining its possible integration into dreams and the brain’s acidity levels during this particularly dynamic sleep stage [48,49,50].

### 2.3. EEG Data

EEG signals were acquired using 19 Ag/AgCl scalp electrodes positioned according to the international 10–20 system with the EEGA-21/26 “Encephalan-131-03” system [51]. Reference electrodes were placed on both the left (A1) and right (A2) mastoids, ensuring that electrode impedance remained below 10 KΩ to maintain high-quality signals. Preprocessing and analysis were conducted in MATLAB (2022b) utilizing the Brainstorm toolbox [52]. The recordings were re-referenced to the linked mastoids to minimize common-mode interference, and artifacts such as eye blinks and other disturbances were removed using Fast Independent Components Analysis [53]. A visual inspection of all signals was performed, and segments affected by muscle activity or non-physiological noise were discarded. To further improve signal quality, a Butterworth band-pass filter (0.5–70 Hz) and a notch filter were employed to eliminate slow drifts, high-frequency noise, and power-line artifacts. The cleaned EEG data were subsequently divided into non-overlapping 2 s epochs to capture the rapid neural characterization of sleep stage transitions.

### 2.4. Statistical Analysis

**Figure 2:**
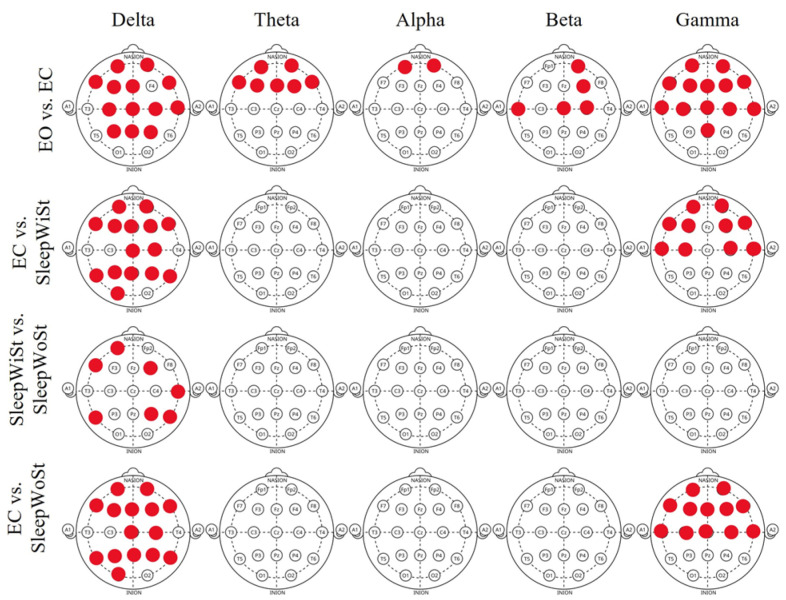
Significant differences ($p_{value}<0.05$) between the experimental conditions (eyes-open vs. eyes-closed, eyes-closed vs. REM sleep with stimuli, REM sleep with stimuli vs. REM sleep without stimuli, and eyes-closed vs. REM sleep without stimuli) are presented for each EEG channel, averaged across all participants. Red markers indicate channels showing statistically significant differences within the corresponding EEG sub-bands.

**Figure 3:**
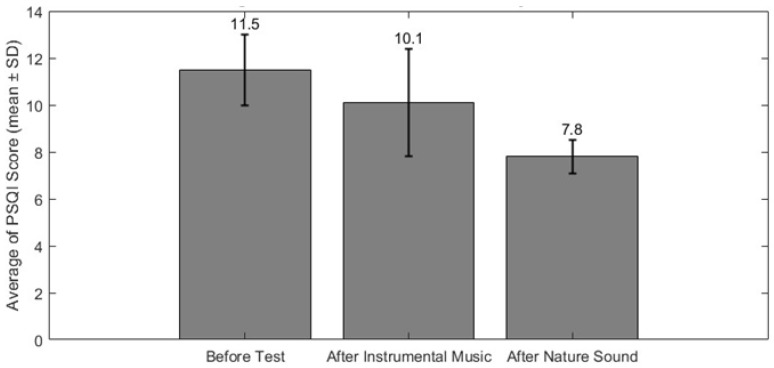
Average PSQI scores before (pre-test) and after the intervention, showing participants’ self-reported sleep quality. Both instrumental and natural auditory stimuli were presented during REM sleep sessions. Error bars indicate standard deviation across participants.

We employed the Kolmogorov–Smirnov test [54] to assess the normality of our data. In this test, a probability value less than 0.05 indicated a non-normal data distribution, while a value greater than or equal to 0.05 suggested normality. Based on those results, the Mann–Whitney U test, a non-parametric test, was chosen to compare differences between various states of sleep. Here, *p*-values lower than 0.05 indicated significant differences between the medians of the features. Figure 2 illustrates the *p*-value for each EEG sub-band analysis on each electrode for wakefulness, NREM, and two different phases of REM sleep. Based on this figure, Fp1, Fp2, F7, P4, T5, and T6 in the delta band show the most considerable influence across different states of EEG recordings. Moreover, Fp1, Fp2, F7, F8, F3, F4, T3, T4, C3, and C4 exhibit significant differences in the gamma band. This indicates that the presence or absence of auditory stimuli impacts the prefrontal, pre-motor, and auditory regions during sleep. However, other EEG frequency bands like theta, alpha, and beta do not show notable differences apart from the comparison between the eyes-open and eyes-closed states. Figure 3 illustrates the enhancement of overall sleep quality before and after exposure to instrumental and natural sound stimuli. The post hoc *t*-test (with an adjusted alpha level of 0.021) revealed that nature sounds resulted in significantly higher PSQI scores ($t=3.56;\;\;p_{value}<0.01$) compared to instrumental music ($t=2.38;\;\;p_{value}<0.01$).

### 2.5. Procedural Framework

**Figure 4:**
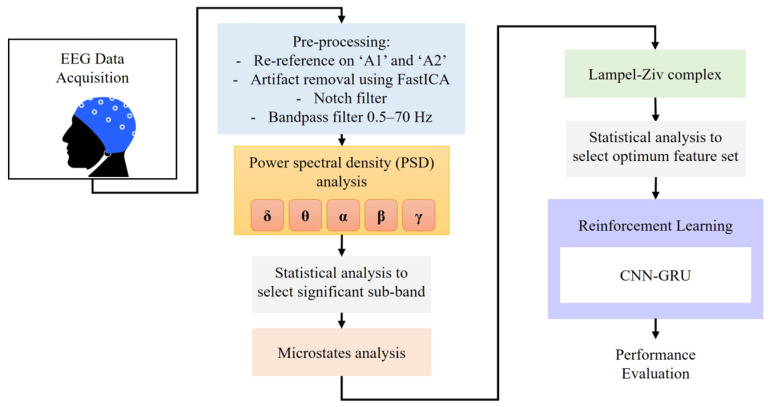
Overview of the EEG data processing and analysis pipeline. EEG signals were initially acquired and pre-processed, which included re-referencing to ‘A1’ and ‘A2’, artifact removal using FastICA, notch filtering, and bandpass filtering (0.5–70 Hz). Power spectral density (PSD) analysis was conducted across δ, θ, α, β, and γ bands, followed by statistical analysis to identify significant sub-bands. Microstate analysis was performed on the selected features. Simultaneously, features were extracted using the Lempel–Ziv complexity method, and optimal feature sets were determined through statistical analysis. The resulting features were then input into a CNN-GRU model optimized with reinforcement learning, and the model’s performance was subsequently evaluated.

Figure 4 presents the schematic block diagram of the proposed procedural classification algorithm. The objective is to assess neuronal dynamic changes during sleep utilizing reinforcement learning. This process encompasses preprocessing, power spectral density (PSD) analysis, statistical analysis to identify effective EEG sub-bands, EEG microstate analysis (including duration, transition, occurrence, and global field power), Lempel–Ziv complexity extraction, and the selection of a significant and optimal feature set of microstates and Lempel–Ziv complexity. Classification is conducted based on a novel structure of reinforcement learning (see Table A1 in the Appendix A).

### 2.6. Biomimetic Inspiration and Algorithmic Parallels

Reinforcement Learning has seen growing application in healthcare and signal analysis due to its capacity to learn optimal policies from sequential data [34,55]. In this study, we utilize RL to enhance EEG-based sleep stage classification, allowing the model to effectively capture temporal dependencies in functional connectivity features. This approach offers distinct advantages over traditional classifiers, such as Support Vector Machine (SVM) and Random Forest (RF), which do not explicitly model sequential interactions [56].

The proposed RL framework draws inspiration from biomimetic principles that replicate the adaptive learning processes found in neural systems [57]. In biological terms, learning is influenced by reinforcement signals, primarily mediated by neuromodulators like dopamine, which encode reward prediction errors and promote behavioral adaptation [58]. Similarly, the RL agent updates its policy based on reward feedback, adjusting its actions to maximize cumulative outcomes [34,59].

The exploration–exploitation trade-off incorporated in our model reflects the balance between novelty-seeking and goal-directed behavior observed in both human and animal learning [60]. Additionally, the reward structure, offering positive reinforcement for accurate classifications and imposing penalties for errors, mirrors the synaptic strengthening and weakening processes that underlie experience-dependent plasticity [61].

By computationally modeling these biologically inspired mechanisms, our approach establishes a biomimetic connection between cortical adaptation and algorithmic learning. This alignment enhances the theoretical foundation of the proposed RL-based classifier for sleep state recognition, bridging neurophysiological insights with adaptive machine intelligence.

## 3. Simulation Results and Discussion

### 3.1. Power Spectral Density (PSD) Analysis

Spectral analysis is one of the best-known methods to describe EEG signals with rhythmic components and has a long history in sleep [62]. It provides a broad overview of signal properties such as signal quality, spectral components, and their variability to make experimental contrasts between different states. Here, the Welch periodogram [63] was used to compute the power spectral density (PSD) for all EEG states. The Welch power spectrum is expressed from the power spectral density as follows [64]:

(1) $$PSD\left(f\right)=\frac{1}{MU}{\left|\sum_{n\;=\;0}^{M-1}x_{i}\left(n\right)\;w\left(n\right)\;e^{-j2\pi f}\right|}^{2},$$

(2) $${PSD}_{Welch}\left(f\right)=\frac{1}{L}\sum_{i=0}^{L-1}PSD(f),$$

where $x_{i}\left(n\right)$ is the EEG sequence, $w\left(n\right)$ stands for windowed data, M is the interval length, and U indicates the normalization factor for power in the window function.

**Figure 5:**
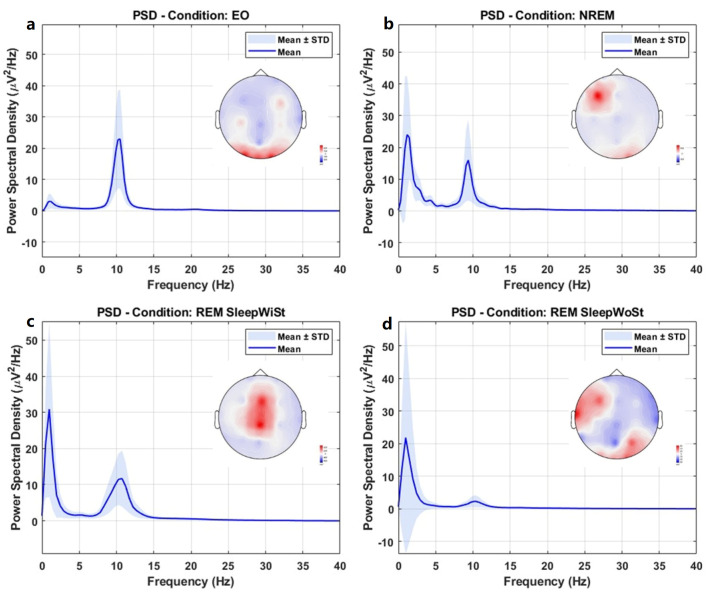
Power spectral density (PSD) curves and topography maps of (**a**) EO, (**b**) NREM or EC, (**c**) REM sleep with stimuli, and (**d**) REM sleep without stimuli. EEG PSD results for the eyes-open condition. (**a**) Group-averaged PSD spectra (mean ± SEM) across all electrodes. (**b**) Topographical distribution of PSD values (µV^2^/Hz) averaged across the alpha frequency band (8–12 Hz). Note: Scales are normalized and kept consistent across conditions to facilitate comparisons.

Continuous EEG data were collected from each subject during all stages. A window size of 2 s was chosen for spectral power analysis to capture rapid variations in brain activity, ensuring high temporal resolution. Although this duration is short, it effectively monitors fluctuations in EEG signals, especially during sleep transitions. Power spectral density (PSD) calculations were conducted on each window, which consisted of 250 EEG samples without any overlap. In Figure 5, the average of the Welch PSD is plotted for the four different stages across all subjects. The corresponding topography maps for each state are shown in the lower panel of this figure. By considering these two panels simultaneously, it can be observed that alpha band power is at its highest value during the EO state, which is mostly related to the *O1* and *O2* electrodes in the visual cortex [65,66,67]. There is also some delta band activation, which may be associated with cognitive processes during the recording period. During the NREM or EC state, alpha band power decreases in the visual cortex, while delta band power increases in the frontal region. In the REM sleep state with stimuli, the PSD of the alpha band is elevated compared to the eyes-closed state within the frequency range of 8–12 Hz, where delta band power significantly increases. As illustrated in the topography map, the most active brain areas are in the central cortex, which are responsible for higher-order processing. These findings are consistent with those of a previous study on lower-frequency activities during REM sleep [68]. When the stimuli are discontinued, the PSD of the delta and alpha bands remarkably decreases, revealing diverse changing patterns from central dominance to the left temporal and right occipital regions, which are related to bottom-up processing in neural oscillations. The Mann–Whitney U test shows significant differences ($p_{value}<0.05$) between the PSD of eyes-open and eyes-closed ($\eta^{2}=0.08;\;\;p_{value}=0.02$), eyes-open and REM sleep with stimuli ($\eta^{2}=0.15;\;\;p<0.05$), eyes-open and REM sleep without stimuli ($\eta^{2}=0.09;\;\;p_{value}<0.05$), and eyes-closed and REM sleep with stimuli ($\eta^{2}=0.06;\;\;p_{value}=0.04$). Nonetheless, there are no major variations between EC and REM sleep in the absence of stimuli. Based on the findings presented in Figure 5, which illustrate the remarkable changes in delta band activity across various states, we conducted additional analysis focusing on delta band power.

### 3.2. EEG Microstate (MS)

EEG microstate analysis is a powerful tool for studying the temporal and spatial dynamics of human brain activity [69]. Brain activity unfolds as a sequence of organized scalp potential topographies, known as microstates, that remain quasi-stable for brief periods (~60–120 ms) before rapidly transitioning to a new configuration [70]. These microstates are believed to arise from the coordinated activity of distributed neural assemblies, generating distinct and reproducible spatial maps in a systematic manner. Consequently, a shift in a topographic map indicates a reorganization in the distribution of the underlying active cortical sources that contribute to the observed potential [71]. The dynamic transitions between successive microstates reflect the temporal sequencing of neural network activations throughout the brain. In contrast to spectral measures that are confined to specific frequency bands, microstates encapsulate the integrative spatiotemporal organization of brain activity, providing valuable insights into the coordination and stability of large-scale neuronal processes during both resting and task-related states [72].

**Figure 6:**
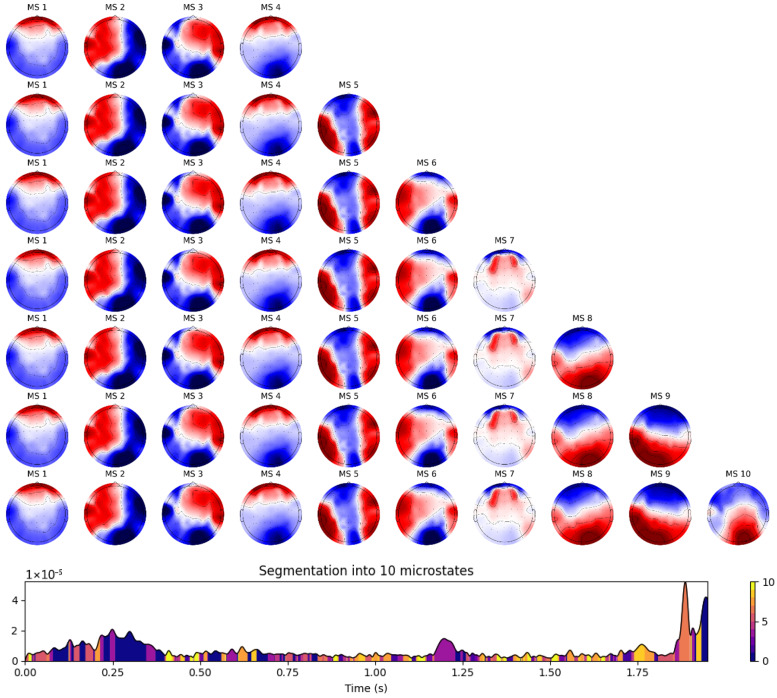
An example of EEG microstate classes from 4 to 10 clusters generated by MNE during REM sleep with stimuli. The K-means clustering analysis method was used to analyze topographic maps. The lower panel shows the back-fitting result for each segment of microstates on GFP signal.

**Figure 7:**
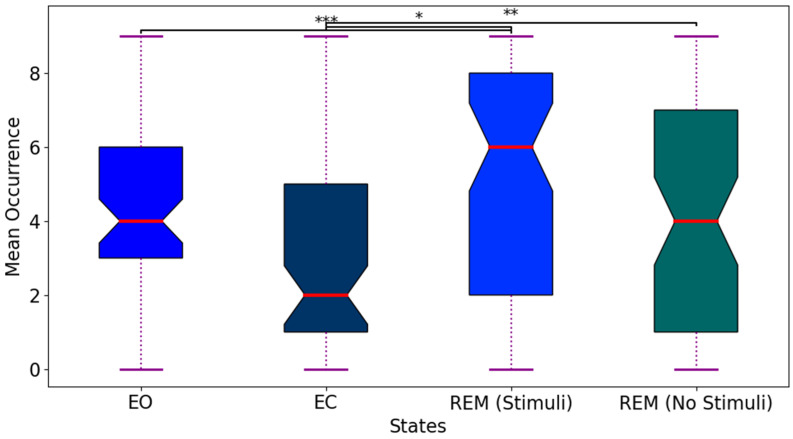
Mean occurrence of EEG microstate classes across sleep states. Boxplots show the interquartile range (first to third quantile), median (red line), and whiskers extending to minimum/maximum values (excluding outliers). Significant differences are indicated: *** $p_{value}<0.001$; ** $p_{value}<0.01$; * $p_{value}<0.05$ (post hoc tests with Bonferroni correction). REM sleep with stimuli exhibits markedly higher occurrence than REM sleep without stimuli.

The pre-processed EEG data was analyzed using the MNE library [73] to detect EEG microstates and compute their characteristics. A standard procedure for EEG microstate analysis involves four stages [74,75,76,77,78]: (1) candidate topography extraction, (2) EEG microstate detection, (3) EEG microstate segmentation, and (4) microstate feature extraction. In the first stage, the global field power (GFP) is employed to characterize the global pattern of neuroelectrical and dynamic fluctuations in the brain, which is defined as follows [74]:

(3) $$GFP\left(t\right)=\sqrt{\left(\sum_{i}^{N}{\left(x_{i}\left(t\right)-\bar{x}\left(t\right)\right)}^{2}/N\right)},$$

where $x_{i}\left(t\right)$ and $\bar{x}\left(t\right)$ are the instantaneous and mean potentials across *N* electrodes at time *t*. In the next stage, the topographies of each peak in the local GFP point are identified to derive successive microstates. In the third stage, all microstates are determined based on microstate patterns using clustering methods. Several studies utilize the K-means clustering method along with the cross-validation metric to demonstrate that the optimal number of classes within subjects is four [76,79]. However, we set the number of clusters from 4 to 10, selecting the optimal set of classes based on the maximum values of global explained variance (GEV). The four well-known topographies are class A (right frontal, left posterior), class B (left frontal, right posterior), class C (midline frontal–occipital), and class D (midline frontal) [78]. Research has shown that each topography remains quasi-stable for approximately 60–120 ms before dynamically transitioning to another pattern. Finally, EEG data was analyzed using a set of topographies that fluctuate dynamically among themselves at discrete time points. The class-labeled maps of REM sleep states were created as a schema to assign the original individual successive series from each subject to 4 to 10 microstate patterns, as shown in Figure 6. Four types of temporal parameters were then computed from each microstate: mean GFP (the average of GFP for a state), duration (the average length of states per unit), occurrence (the average frequency of detected states), and coverage (the percentage of each state appearing in every epoch). Figure 7 depicts the average occurrence of microstates during EO, EC, REM sleep with stimuli, and REM sleep without stimuli across all participants. According to the GEV values for different numbers of EEG microstate classes (see Table 1), the optimal number of microstates was determined, and their labels were organized into a sequence using the modified k-means clustering algorithm and GEV criteria. GEV measures the similarity of each EEG sample to the microstate prototype, with higher GEV values indicating optimal assignment. The maximum GEV value was selected after 10 iterations of re-running the analysis.

### 3.3. Lempel–Ziv Complexity (LZC)

**Figure 8:**
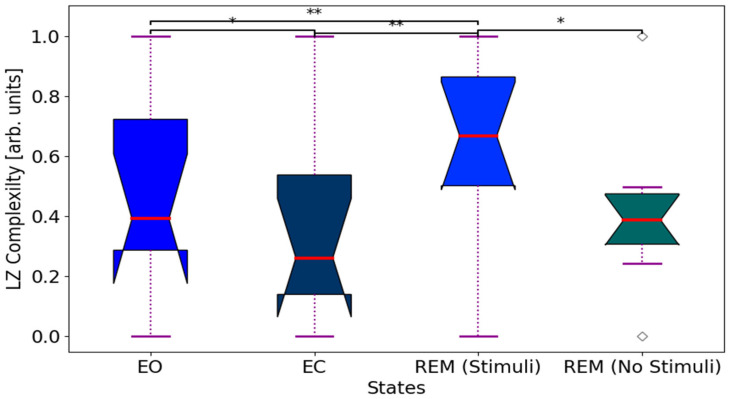
Lempel–Ziv complexity dynamics across sleep states. LZC markedly increases from EC to REM sleep with auditory stimuli, then significantly decreases in REM sleep without stimuli, reflecting heightened brain signal complexity under stimulation. Boxplots display the interquartile range (first to third quartile), median (red line), and whiskers extending to minimum/maximum values (outliers excluded). Post hoc pairwise comparisons (Bonferroni-corrected): ** $p_{value}<0.01$; * $p_{value}<0.05$.

The LCZ quantifies the regularity of a time series through its frequency factors [80]. LZCs were derived from the temporary characteristics of microstates across each channel. This analysis depended on the complex-valued analytical representation of the data. In this framework, the original microstate acts as the real part, while the Hilbert-transformed signal serves as the imaginary part. The analytical signal can be represented as the product of a time-dependent real-valued positive amplitude and complex-valued phase angle. The analytical signal was converted into a binary format by implementing a threshold based on the median amplitude value within the specified window, and the complexity or compressibility of the resultant binary string was evaluated using the LZC algorithm [81]. We noted a significant reduction in complexity ($p_{value}<0.05$) when comparing REM sleep with stimuli to REM sleep without stimuli (Figure 8). Increasing LZC values from NREM to REM sleep with stimuli could be due to reflecting higher cognitive engagement. In addition, LZC differed between the two resting states (EO and EC), likely indicating a difference in microstate occurrence. This analysis supports the neuronal dynamics oscillatory components exert a greater influence on LZC during the sleep states.

### 3.4. Reinforcement Learning Classifier

Our classification model for sleep state detection is classified into four key components: (1) an attention-based CNN-reinforcement learning architecture, (2) hierarchical feature integration, (3) an end-to-end optimization strategy, and (4) task-specific applications in EEG signal classification. First, the model uses GFP signals to guide attention across brain regions, with a CNN extracting local features and an RL agent dynamically integrating them into global representations. Second, feature processing hierarchically aggregates temporal–spatial patterns, enabling the model to focus on task-relevant regions. Third, optimization employs end-to-end training, where backpropagation updates CNN weights and policy gradients adjust the GRU-RL agent’s decisions [82].

In the proposed RL framework, the *environment* represents the evolving neural dynamics observed in the EEG signals during different sleep states. In each time step t, the *environmental state* $s_{t}$ is defined as a multimodal feature vector that combines the GFP amplitude sequence with temporal microstate parameters and LZC value extracted from the same segment. This composition allows the agent to perceive both global synchronization (via GFP) and local neural variability (via microstates and LZC), reflecting the underlying physiological transitions across sleep stages. The *action space* $a_{t}$ corresponds to classification decisions identifying the current sleep state (EO, EC, REM with stimuli, REM without stimuli). The *feedback (reward) function* $r_{t}$ provides positive reinforcement for correct decisions and small penalties for incorrect predictions, encouraging adaptive learning of temporal dependencies.

The mapping between the environmental state and the CNN + GRU hidden state represents how the agent encodes EEG-derived information over time, capturing the gradual evolution of sleep dynamics. Thus, the RL agent’s policy is driven by observing changes in microstates and LZC features, emulating the adaptive mechanisms of biological decision-making during neural state transitions.

**Figure 9:**
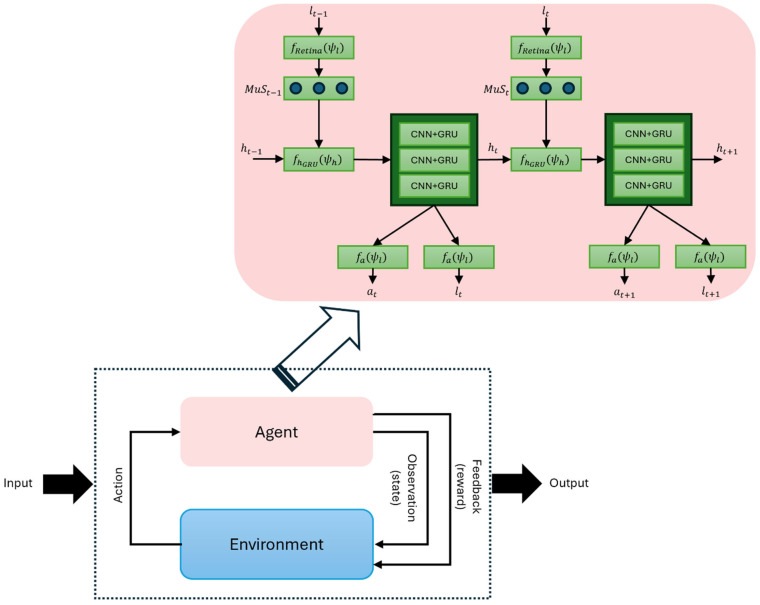
Architecture of the proposed reinforcement learning model. The agent interacts with the environment by observing sample signal segments through multi-scale receptors. In each time step, the environment provides a sequence, and the agent extracts multi-resolution features around a selected location. These features constitute the internal state, which is refined using a CNN + GRU network. The agent produces two actions: (1) selecting the subsequent signal location for sampling, and (2) predicting the state of sleep. Following each action, the environment provides a reward of 1 for accurate classification and 0 if incorrect. The agent optimizes its policy by fine-tuning its parameters to enhance cumulative rewards through the process of reinforcement learning.

An important feature of the proposed framework is that the number of trainable parameters and overall computation can be regulated independently of the input data size, unlike conventional deep networks. In our design, the computational cost of the CNN + GRU component increases linearly with both the signal length and the number of EEG channels. Figure 9 illustrates the attention-driven reinforcement learning architecture, where CNN + GRU serves as the fundamental network and agent. In each iteration t, the agent perceives the environment through the GFP sequence, $x_{t}$. Rather than processing the full input at once, the agent extracts information using localized receptors with a restricted field of view, $f_{Retina}$, thereby sampling only portions of the GFP sequence. Additionally, multi-scale sensors, ${MuS}_{t}$, are employed to capture task-relevant temporal dynamics at different time resolutions. These receptors operate around a central location, $l_{t-1}$, but also consider surrounding positions, with spatial resolution highest near the center and gradually decreasing at more distant points. This hierarchical structure is formally defined in Equation (4), where $\psi_{MuS}$ represents the parameter growing the multi-scale neural network:

(4) $${MuS}_{t}=F_{MuS}(x_{t},l_{t-1},\;\psi_{MuS}).$$

The agent keeps a sequence of states at every time step that maps environmental data gathered from ${MuS}_{t}$ sensors. As a result, GFP signals are interpreted into environmental knowledge, which is then utilized as input for agent action neural networks to produce actions. This allows it to focus on specific GFP segments in specific locations, achieving an attention mechanism with high selectivity. The agent’s state sequence is created by the hidden unit $h_{t}$ of the underlying CNN + GRU, and each step is dynamically updated as follows to act as the input for the CNN + GRU component:

(5) $$h_{t}=f_{h\_GRU}\left(h_{t-1},{MuS}_{t}:\;\psi_{h}\right).$$

The action that the agent must perform is carried out by a localization neural network, which identifies the sampling position $l_{t}$ of ${MuS}_{t}$ through this neural network. In our framework, the locations are chosen at random from the parameterized distribution of the location network $f_{l}\left(h_{t}:\psi_{h}\right)$ at time t:

(6) $$l_{t}\approx p\left(.|f_{l})(h_{t\_out}:\psi_{l}\right).$$

Here, $f_{l}$ and $\psi_{l}$ refer to the neural network’s position and its corresponding parameters. Likewise, the actions taken by the environment are derived from a distribution that is conditional on the output of the action network, $a_{t}\approx p\left(.|f_{a})(h_{t\_out}:\psi_{a}\right)$, where $f_{a}$ and $\psi_{a}$ denote the neural network that produces actions and the parameters of that network. The reward is denoted as $R=\sum_{t=1}^{T}\gamma^{t-1}r^{t}$ and $r^{t}=1$ if the GFP signal is correctly classified at time t and $r^{t}=0$ otherwise. The model introduced in this paper deals with a partially observed Markov decision process, with the agent’s learning goal being a stochastic strategy, P, characterized by the parameter ψ.

(7) $$P=\pi \left(\left(l_{t},a_{t}\right)|s_{1:t};\psi \right).$$

In Equation (7), π represents the strategy function and $s_{1:t}=x_{1},\;l_{1},\;a_{1},\;\ldots ,\;x_{t-1},\;l_{t-1},\;a_{t-1},\;\ldots .,\;x_{t},$ defines the interaction mapping between the agent and the environment. By persistently refining the parameters $\psi =\left\{\psi_{MuS},\;{\psi_{h},\;\psi }_{a}\right\}$ associated with MuS sensor, core network, and action network to enhance cumulative rewards, we characterize the ongoing optimization of strategies by intelligent agents aimed at maximizing returns as below:

(8) $$J\left(\psi \right)=E_{p(s_{1:T};\;\psi )}\left[\sum_{t=1}^{T}r_{t}\right]=E_{p(s_{1:T};\;\psi )}\left[R\right].$$

The precise solution to Equation (8) can be presented as follows:

(9) $$\nabla_{\psi }J=\sum_{t=1}^{T}E_{p(s_{1:T};\;\psi )}\left[\nabla_{\psi }\log\pi \left(u_{t}|s_{1:t};\;\psi \right)\;R\right]\approx \frac{1}{M}\sum_{i=1}^{M}\sum_{t=1}^{T}\nabla_{\psi }\log\pi \left(u_{t}^{i}|s_{1:t}^{i};\;\psi \right)\;R^{i}.$$

Here, $s_{1:t}^{i}$ represents the state of the CNN + GRU hidden unit in i=1, …, M, and the gradient of ψ is determined through conventional gradient backpropagation [83]. Nonetheless, there might be a significant squared difference between the equation above and the state value function, $E_{\pi }\left[R\right]$ [84], which can be identified by optimization [84] as in the below equation:

(10) $$\frac{1}{M}\sum_{i=1}^{M}\sum_{t=1}^{T}\nabla_{\psi }\log\pi \left(u_{t}^{i}|s_{1:t}^{i};\;\psi \right)\left(\;R_{t}^{i}-E_{\pi }\left[R_{t}\right]\right).$$

The value function is crucial for smoothing expected returns based on past data, balancing exploration, directing strategy, and incorporating reinforcement learning to mitigate high variance.

The RL classifier was configured with optimized hyperparameters to ensure convergence and stability in sleep stage classification. The state space was defined based on the feature space, which comprised microstate parameters and LZC values. The action space was characterized by classification decisions corresponding to wakefulness, NREM, REM sleep with stimuli, and REM sleep without stimuli. The reward function was fed by positive reinforcement for correct classifications and a minor penalty for incorrect ones, promoting adaptive decision-making. The ε-greedy exploration strategy was implemented with ε decaying from 1.0 to 0.1 over 1000 episodes. The learning rate was set to 0.001, and a discount factor (γ) of 0.9 was used to balance immediate and future rewards. Training occurred over 500 epochs with a batch size of 128, utilizing the Adam optimizer. To mitigate overfitting, early stopping and a dropout rate of 0.2 were applied. All computations were executed on a Windows 11 system equipped with an Intel Core i7 processor, 32 GB of RAM, and an NVIDIA RTX 1080 Ti GPU. These settings align with reinforcement learning practices within the EEG classification literature and were tuned to achieve optimal model generalization and stability.

## 4. Experimental Design

### 4.1. Experimental Setup

Three experiments are conducted to assess the performance of the proposed model based on the microstates and LZC features. The first experiment concerned the evaluation of the sleep state detection procedure using different features extracted by the microstate and LZC, separately. Then, the optimal feature set in terms of accuracy was selected to use in the next step. In the second experiment, the efficiency of different deep RL models was evaluated with an optimal feature set. Finally, we chose the best set of features and classifiers to categorize sleep states with various numbers of EEG channels. This step was performed because of the importance of using fewer electrode numbers during sleep.

To conduct the first experiment, the EEG data of 33 subjects during the four states were selected to analyze the performance of the deep classification model. Temporary microstate parameters and LZC features were obtained on the EEG signals on a non-overlap window of 256 samples. The extracted features are given to the three different structures of the proposed model (i.e., CNN-RL, GRU-RL, and CNN + GRU-RL) for classifying wakefulness, NREM, REM with stimuli, and REM without stimuli. Here, 75% of the data was used as a training set, and the rest was considered the test set. In the second experiment, the combinations of MS and LZC as multivariate feature analysis were fed to the classifiers. This was performed to find appropriate features with high performance in sleep states classification from EEG signals. To account for stochasticity in RL training, each experiment was repeated 10 times using k-fold cross-validation with different random initializations, and the average accuracy ± standard deviation was reported.

The performance of the proposed model was compared with EEGNet [85] to evaluate its efficiency in the optimal structure. In addition, to assess the generalizability and robustness of our method, we utilized an external dataset from [40]. This dataset was recorded from 28 healthy male participants without any reported physiological or sleep disorders. It contains five sleep stages labeled according to AASM criteria: wake, N1 (lightest stage), N2 (intermediate stage), N3 (deepest stage), and REM, each annotated in 30 s epochs (see [40] for details).

To maintain consistency in our experimental design and feature extraction procedure, four 30 s epochs from the same stage were concatenated to create continuous recordings for each class. This methodology enabled multi-scale feature extraction using various window sizes identical to those applied in our experimental data. The external dataset was utilized exclusively as a benchmark to assess the generalization capability and classification superiority of the proposed model, rather than for a direct one-to-one comparison with our aggregated NREM condition.

### 4.2. Evaluation Metrics

The effectiveness of the proposed classification algorithm is assessed using three metrics: accuracy, positive predictive value (PPV), and negative predictive value (NPV) [86]. The accuracy (ACC) metric indicates the overall correctness of detection. The PPV metric represents the proportion of true positive test results that are indeed positive, while the NPV metric measures the proportion of true negative results that are indeed negative. In this study, TP, FP, TN, and FN denote true positives, false positives, true negatives, and false negatives predicted by the algorithm, respectively.

(11) $$ACC=\frac{TP+TN}{TP+TN+FP+FN},$$

(12) $$PPV=\frac{TP}{TP+FP},\;$$

(13) $$NPV=\frac{TN}{TN+FN}.$$

## 5. Results and Analysis

To delineate the optimal procedure for classifying sleep states using an attention-based RL model, a statistical analysis of both single and multivariate features across states was conducted, followed by an evaluation of the impact of various deep model architectures. The optimal feature set was identified based on significance values ($p_{value}<0.05$). Subsequently, this optimal feature set was input into the attention-based RL model with diverse architectures to assess the effectiveness of the classification algorithm.

### 5.1. Statistical Analysis

The Mann–Whitney U test [87] was used to compare differences between two states, as demonstrated by the Kolmogorov–Smirnov test [54], which indicated a non-normal distribution across these states ($p_{value}<0.05$). Table 2 and Table 3 present the significant *p*-values for each individual microstate temporal parameter and their combinations with the LZC feature extracted from the delta band. According to these results, the computed LZC from occurrence data reveals significant differences between sleep states in general.

### 5.2. Classification Performance

**Figure 10:**
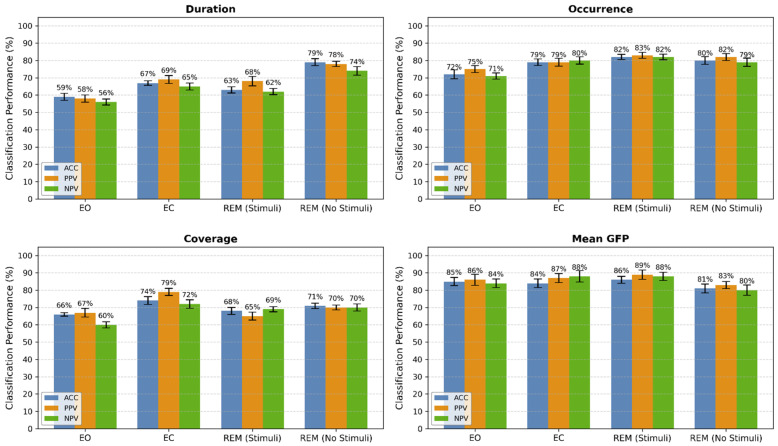
Classification performance (mean ± SD) of GRU-RL using only microstate features across sleep states. Bars represent accuracy (ACC, blue), precision (PPV, orange), and negative predictive value (NPV, green). Error bars indicate standard deviation.

**Figure 11:**
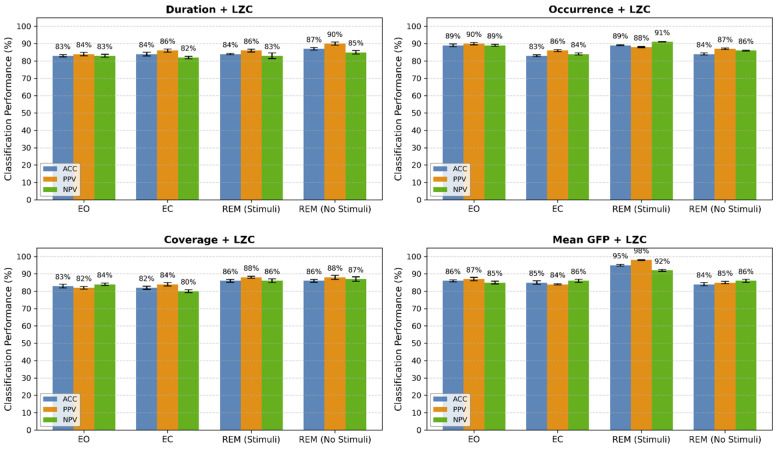
Classification performance (mean ± SD) of GRU-RL using pairwise combinations of each microstate feature with LZC across sleep states. Bars represent accuracy (ACC, blue), precision (PPV, orange), and negative predictive value (NPV, green). Error bars indicate standard deviation.

The results of employing the GRU-RL classifier are presented in Figure 10, which details each microstate feature: duration, occurrence, coverage, and mean GFP. The results include ACC, PPV, and NPV for delta band signals segmented into 2 s intervals. These parameters demonstrate a satisfactory classification performance, with ACC values of 67 ± 1.78%, 78.25 ± 2.0%, 69.75 ± 1.7%, and 84 ± 2.3% corresponding to duration, occurrence, coverage, and mean GFP across four states. It is noted that the highest ACC was achieved in the “mean GFP” compared to the other microstate parameters. To enhance classification performance, the optimal microstate feature was selected based on classification accuracy. Figure 11 displays the detection of sleep states through the classification of multivariate features. In this analysis, Lempel–Ziv complexity was extracted from occurrence, duration, coverage, and mean GFP for each of the states: EC, EO, REM sleep with stimuli, and REM sleep without stimuli. According to the figure, the highest ACC of 87.5 ± 0.75% was attained using the “mean GFP + LZC” feature set.

**Figure 12:**
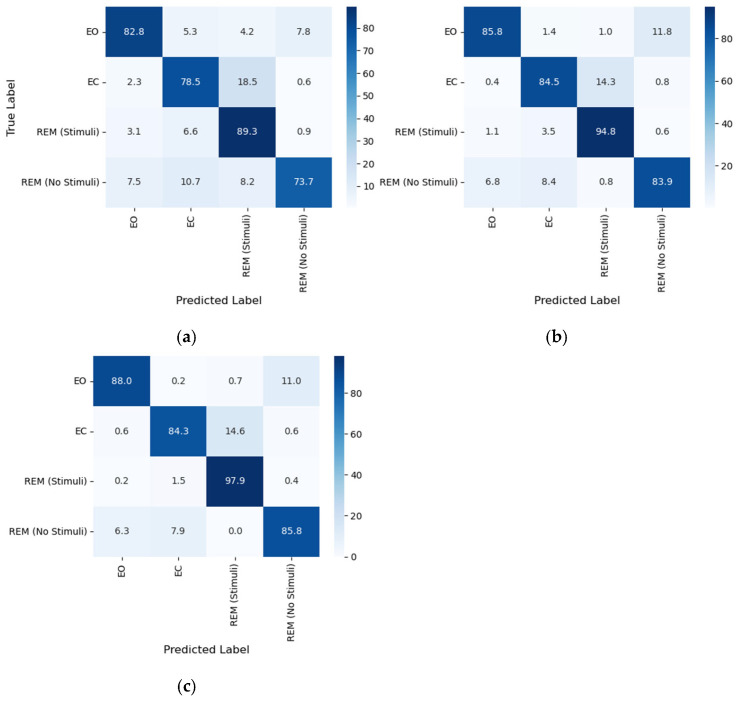
Confusion matrices for three model architectures, (**a**) CNN-RL, (**b**) GRU-RL, and (**c**) CNN + GRU-RL, using the optimal feature combination of “mean GFP + LZC”. The models classified four sleep states: wakefulness (EO), NREM (EC), REM with stimuli, and REM without stimuli. The hybrid CNN + GRU-RL structure achieved the highest overall accuracy, with superior discrimination across all classes, particularly in REM conditions.

In a subsequent experiment, the performance of the proposed classifier was evaluated using various structures as depicted in Figure 12. For this purpose, the optimal feature set was provided to the CNN-RL (a), GRU-RL (b), and CNN + GRU-RL (c) models. The average classification performance of the proposed model is illustrated in this figure. Notably, the detection performance of the proposed algorithm shows a significant improvement with the addition of the GRU layer to the deep RL model. This enhancement may be beneficial for real-time applications in detecting sleep disorders.

**Figure 13:**
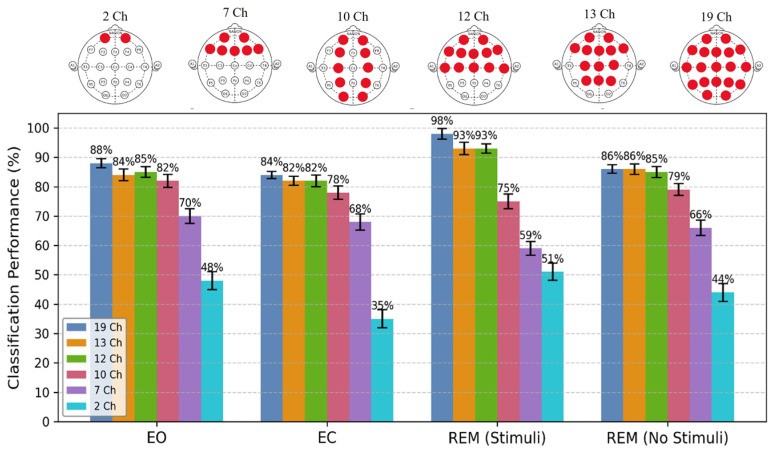
Impact of EEG channel selection on sleep stage classification accuracy (mean ± SD) using “mean GFP + LZC” features and the proposed CNN + GRU-RL model. Performance was evaluated across channel subsets: full 19-channel montage (dark blue), 13 channels (orange), 12 channels (green), 10 channels (purple), 7 channels (pink), and 2 channels (light blue). The 19-channel configuration achieved peak accuracy (88–98% across states), with REM (Stimuli) reaching 98%. Accuracy remained robust (>80%) with 12–13 channels, especially in REM conditions. The 7-channel setups retained above 60% accuracy in most states, demonstrating strong generalization and resilience to reduced sensor input. Error bars indicate standard deviation.

Figure 13 presents a comparison of the sleep state classification performance (mean ± standard deviation) of the proposed method, which employed the optimal feature set (i.e., mean GFP + LZC) and the GRU+CNN-RL classifier, using various EEG electrodes in terms of ACC measures. The results indicate that the accuracy of sleep state detection generally decreased as the number of EEG channels was reduced. The deep learning model demonstrated superior performance when utilizing all 19 channels, achieving accuracy rates of 88%, 84%, 98%, and 86% for EC, EO, REM sleep with stimuli, and REM sleep without stimuli, respectively.

However, the model maintains high performance with 13 and 12 selected channels from the frontal and central lobes, yielding accuracy rates of 84 ± 2.0%, 82 ± 1.5%, 93 ± 2.1%, 86 ± 1.8%, and 85 ± 1.8%, 82 ± 2.0%, 93 ± 1.6%, and 85 ± 1.9% for the same states. This suggests that EEG channels located in the frontal and central regions can effectively detect sleep states, performing comparably to using all EEG cap channels. Reducing the number of EEG electrodes not only lowers costs and enhances subject comfort but also decreases computational time for real-time data processing. It is noteworthy that classification results for two channels fall below chance level, with accuracy rates of 48 ± 3.0%, 35 ± 3.1%, 51 ± 2.9%, and 44 ± 3.0% for EO, EC, REM sleep with stimuli, and REM sleep without stimuli, respectively. Therefore, it seems that at least seven EEG channels are required to achieve performance that exceeds chance level, with classification accuracies of 70%, 68%, 59%, and 66% for EO, EC, REM sleep with and without stimuli.

In the final experiment, we aimed to compare the proposed classification algorithm with the RF [88], SVM [89], CNN [90], GRU [27], and EEGNet [85] classification models and the dataset by Höhn et al. [40]. The results presented in Table 4 show that the accuracy of the classification models decreased as the time window length increased. Nonetheless, the SVM and GRU classifiers demonstrated suitability for long-term data analysis.

The proposed CNN + GRU-RL model achieved the highest accuracy of 97.2% when analyzing 1 s segment EEG data. This finding suggests that shorter EEG signal lengths in microstate and complexity analysis have a more significant impact on differentiating brain function within milliseconds. Furthermore, the proposed RL model outperformed the EEGNet [85] classification algorithm, which is an open-source toolbox based on a CNN architecture. Additionally, the application of the CNN + GRU-RL classifier to sleep data from [40] demonstrated strong generalization ability, achieving an accuracy of 93.1% for sleep state classification with a 1 s window length.

### 5.3. Limitations and Future Work

The findings of this study should be interpreted in light of several limitations. First, the experiments were conducted exclusively on healthy participants, while individuals with sleep disorders may exhibit altered sleep architecture and EEG spectral profiles that could differentially affect microstate dynamics and complexity measures. Second, the auditory stimulation protocol employed identical sequences across all participants without randomization or counterbalancing. Although this approach ensured experimental consistency, it may have introduced systematic bias related to the order of stimuli. Future research should implement randomized or counterbalanced stimulus presentation to better isolate sequence-dependent effects on neural responses during sleep. Finally, although the proposed model effectively classified wakefulness, NREM, and REM stages, with a particular focus on REM responses to auditory stimulation, it did not distinguish between NREM substages (N1, N2, N3). Analyzing these substages separately could reveal stage-specific influences of auditory stimulation on brain dynamics and further refine sleep monitoring applications.

## 6. Conclusions

This research introduces an advanced method for classifying sleep states using microstates and Lempel–Ziv complexity obtained from EEG signals, utilizing reinforcement learning via a CNN + GRU neural network framework. In this research, all subjects were exposed to auditory stimuli, including music and nature sounds, during a brief period of REM sleep, while EEG data were recorded prior to falling asleep, during NREM sleep, and during REM sleep. Initially, microstate analysis was conducted to extract temporal parameters from pre-processed EEG data. Subsequently, Lempel–Ziv complexity was calculated for each microstate parameter to capture the emerging complex behavior of the brain. These features were provided to the GRU-RL classifier in both single and multivariate modes to identify the optimized classification structure. The results indicate that computing LZC from the mean GFP yielded higher accuracy with the CNN + GRU-RL classifier compared to other feature sets and deep model architectures.

Classification performance demonstrates that the proposed sleep state classification algorithm outperforms the EEGNet [85] baseline system and is also generalizable on the dataset developed by Höhn et al. [40]. Furthermore, the proposed algorithm achieves optimal classification performance in a shorter timeframe, which is an advantage for urgent application requirements.

Furthermore, the network accepts multi-scale windows as input, using the internal state of CNN + GRUs to establish the next focal position and produce control signals in changing environments. Although it is non-differentiable, the suggested unified architecture employs policy gradient techniques for end-to-end training, including both the input and the actions. In EEG signal processing, attention models that integrate reinforcement learning concepts can effectively focus on vital features, automatically eliminate noise and superfluous data, and improve signal decoding precision. This mechanism greatly enhances the model’s resilience and adaptability by consistently fine-tuning parameters and responding flexibly to varying noise levels. Additionally, by actively engaging with dynamic environments, the reinforcement learning-driven attention mechanism continuously hones model performance, learning the unique qualities of each signal. This personalized learning ability improves the model’s capacity to tolerate subjective differences. Moreover, the combination of the CNN and GRU enables accurate detection of intricate temporal relationships in EEG signals while incorporating attention mechanisms. The GRU not only enhances analytical efficacy in the temporal realm but also expands the range of feature extraction, allowing for effective analysis of multidimensional information within both the temporal and spatial arenas of EEG signals.

Experimental findings reveal that the CNN + GRU model utilizing reinforcement learning performs competitively in the multivariate analysis of EEG signal classification tasks in comparison to models relying solely on a CNN or GRU. Furthermore, our proposed algorithm accommodates various fascinating extensions, such as implementing an alternative action role that allows the network to terminate at any moment and make a conclusive classification decision. This positions our approach as a strong competitor in the realm of dynamic EEG processing, alongside well-established models such as the CNN and EEGNet.

## Figures and Tables

**Table 1:** GEV values using K-means clustering for different numbers of *N* after 500 iterations.

Number of Microstates (*N*)	GEV
2	0.5827
3	0.5858
4	0.6139
5	0.6048
6	0.6344
7	0.6787
8	0.7531
9	0.8470
10	0.8615

**Table 2:** *p*-values of the Mann–Whitney test for extracted microstate features from preprocessed EEGs. The symbol * indicates a significant difference ($p_{value}<0.05$).

MS Parameters	Duration	Occurrence	Coverage	Mean GFP
EO vs. EC	0.059	0.137	0.174	0.305
EC vs. SleepWiSt	0.049 *	0.023 *	0.380	0.096
SleepWiSt vs. SleepWoSt	0.026 *	0.016 *	0.332	0.042 *
EC vs. SleepWoSt	0.125	0.114	0.098	0.090

**Table 3:** *p*-values of the Mann–Whitney test for multivariate features (“MS + LZC”) extracted from the EEG. The symbol * indicates a significant difference ($p_{value}<0.05$).

MS + LZC	Duration	Occurrence	Coverage	Mean GFP
EO vs. EC	0.142	0.051	0.061	0.034 *
EC vs. SleepWiSt	0.019 *	0.029 *	0.078	0.221
SleepWiSt vs. SleepWoSt	0.038 *	0.022 *	0.041 *	0.013 *
EC vs. SleepWoSt	0.065	0.015 *	0.020 *	0.051

**Table 4:** Comparison of proposed sleep classification method with baseline algorithms, including Random Forest (RF) [88], Support Vector Machine (SVM) [89], convolutional neural network (CNN) [90], gated recurrent unit (GRU) [27], EEGNet [85], and dataset of [40] in terms of ACC (%) for varying lengths of EEG signal segments.

Time Window	1 s	5 s	10 s	20 s	30 s	40 s
RF [88]	58.7	60.1	64.7	79.1	82.0	78.3
SVM [89]	74.6	86.9	95.5	95.8	96.9	87.1
CNN [90]	71.2	74.4	77.6	81.5	83.7	74.8
GRU [27]	72.1	83.3	85.0	87.7	93.7	90.3
Höhn et al. dataset [40]	93.1	87.5	83.4	78.6	79.2	79.7
EEGNet model [85]	75.7	81.4	76.4	76.6	77.0	71.5
Proposed model	97.2	85.7	84.3	80.5	81.3	78.5

## Data Availability

The data that support the findings of this study are not openly available due to reasons of sensitivity and are available from the corresponding author upon reasonable request.

## Appendix A

Table A1 presents a structured overview of the complete processing workflow, including raw EEG feature extraction, model training using the CNN + GRU_RL architecture, and the cross-subject evaluation protocol.

**Table A1:** Summary of the pseudocode for the proposed CNN + GRU reinforcement learning model in sleep state classification.

**# Step 1: Pseudocode for raw EEG data analysis** **Input**: EEG data as time series (19 channels × time) **Output**: Feature matrix for RL model *Pre-process EEG time series **t**;* *Filter (0.5–70 Hz)* *Remove artifacts* *Segment into time windows* *For each channel ch and each time window ws:* *Compute PSD using the Welch method* *Compute microstate features* *Compute Lempel-Ziv complexity* *Concatenate features into a feature matrix* *Perform significant analysis to identify significant sub-bands/features* *Final output: feature matrix (2×19 D)* **# Step 2: Training of CNN + GRU_RL model** **Input**: feature matrix (**2 × 19** D features), Labels **Output**: Trained CNN + GRU_RL model *Split the training set into training and validation sets* *# Model architecture:* *Input layer: feature matrix with shape (1,38,1)* *Convolutional layers:* *Conv1: 256 filters, kernel size 7, ReLU activation* *Conv2: 64 filters, kernel size 5, ReLU activation* *Conv3: 16 filters, kernel size 3, ReLU activation* *Max Pooling layer: size 3×3* *GRU layer: 256 units* *Fully connected layer: 16 neurons, SoftMax* *Output layer: 4 neurons, linear activation # for RL policy* *Optimizer: Adam with learning rate 0.001* *Loss function: cross entropy* *Training parameters: Batch size 128, epochs 500* *For each epoch = 1 to Total_Epochs (500), do* *Initialize model parameters* $\psi ={\{\psi }_{r},\;\psi_{h},\;\psi_{a},\psi_{l}\}$: *Initialize hidden state* $h_{0}$ *Initialize location* $l_{0}$ *For episode = 1 to maxEpisode do* *Reset environment* t=1 $l_{t}=$ *initial location* $h_{t-1}=h_{0}$ *while not terminal do* *# Retina* ${MuS}_{t}=f_{Retina}(l_{t},\psi_{r})$ *# Feature extraction* via *CNN + GRU* $h_{t}=f_{GRU}({MuS}_{t},{h_{t-1},\psi }_{h})$ *# Attention selection* $a_{t}=f_{a}({h_{t},\psi }_{a})$ *# Next location* $l_{t+1}=f_{l}({h_{t},\psi }_{l})$ *# step in Environment* $\left(state_{next},\;reward_{t},\;done\right)=Environement.step(a_{t})$ *# Store transition and update* $store\;\left(MuS_{t},\;h_{t},\;a_{t},\;reward_{t},\;l_{t},\;l_{t+1}\right)$ update parameters ψ using RL objective *# Move to next time step* $h_{t-1}=h_{t}$ $l_{t}=l_{t+1}$ t=t+1 *if done, then break* *end while* *end for* *end for* *Evaluate the model on the validation set* *Save the best-performing model* **# Step 3: Testing and cross-subject evaluation** *For each subject i:* *Use subject i as the test set* *Train the model on all remaining subjects* *Evaluate the trained policy on the unseen EEG of subject i* *Compute ACC, PPV, NPV* *Aggregate results across subjects* *Return the final trained model and + cross-validation metrics*

For clarity, Table A2 summarizes all model parameters and hyperparameters used in the proposed RL process, along with their functional roles.

**Table A2:** Summary of all parameters and hyperparameters used in the reinforcement learning framework.

$x_{t}$	Signal sequence in each time step t
$f_{Retina}(x_{t},\;l_{t-1})$	Formal representation of a signal $x_{t}$ by location network
${MuS}_{t}$	Multi-scale feature vector
$\psi_{MuS}$	Parameter of the multi-scale neural network
$h_{t}$	Hidden unit
$\psi_{h}$	Parameter of the hidden neural network
p(.)	Parameterized distribution
$f_{l}$ and $\psi_{l}$	Position the neural network and the parameters of the neural network
$f_{a}$, $\psi_{a}$, and $a_{t}$	Action generated by the neural network, the parameter of the neural network, and the generated action at time t formulated by the SoftMax function
$r^{t}$ and R	Reward and cumulative reward
P	Stochastic strategy
$s_{1:t}$	Mapping the interaction between agent and environment
$J\left(\psi \right)$	Optimization function
$s_{1:t}^{i}$	State of CNN + GRU hidden unit in interaction with the environment
$\nabla_{\psi }J$	Gradient of CNN + GRU (standard gradient backpropagation)
$E_{\pi }\left[R\right]$	State value function
